# Anti‐TNF‐induced lupus in patients with inflammatory bowel disease

**DOI:** 10.1002/jgh3.12291

**Published:** 2019-12-19

**Authors:** Sherman Picardo, Kenji So, Kannan Venugopal

**Affiliations:** ^1^ Department of Gastroenterology and Hepatology Royal Perth Hospital Perth Western Australia Australia

**Keywords:** adalimumab, anti‐tumor necrosis factor, anti‐TNF‐induced lupus, inflammatory bowel disease, infliximab, lupus

## Abstract

**Background and Aims:**

Anti‐Tumor Necrosis Factor (TNF)‐induced lupus (ATIL) is a distinct clinical entity, increasingly recognized in patients with inflammatory bowel disease treated with anti‐TNF therapy. Our aims were to evaluate the incidence and clinical and serological markers of ATIL in this population.

**Methods:**

This observational cohort study reviewed 454 patient treatment courses with anti‐TNF therapy (300 infliximab and 154 adalimumab). A diagnosis of ATIL was based on the most widely accepted diagnostic criteria: (i) a temporal relationship between symptoms and anti‐TNF therapy and resolution of symptoms following cessation of the offending medication; (ii) at least one serologic American College of Rheumatology (ACR) criterion of Systemic Lupus Erythematosus (SLE); and (iii) at least one nonserological criterion such as arthritis, serositis, or rash. Clinical, demographic, and serological predictors were evaluated.

**Results:**

The incidence rate of ATIL was 5.7% for infliximab and 0.6% for adalimumab, which are much higher than previously reported postmarketing estimates. The median duration to diagnosis following commencement of anti‐TNF therapy was 15 months (3–62 months). ATIL occurs more commonly patients that commence therapy at an older age (46.47 years ± 13.79 years vs. 38.85 years ± 14.75 years, *P* = 0.033).

**Conclusions:**

ATIL is a significant complication of anti‐TNF therapy, affecting 1 in every 20 patients who commence infliximab. A panel of serological markers is useful to confirm the diagnosis and exclude other conditions that may mimic ATIL. Clinicians using anti‐TNF medications should counsel patients about this potential risk and monitor for clinical manifestations of lupus during routine follow up.

## Graphical abstract

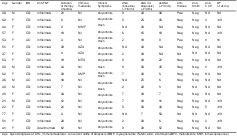

This is a retrospective study that looks at anti‐TNF‐induced lupus, a distinct clinical entity increasingly identified in patients with inflammatory bowel disease on anti‐TNF therapy. ATIL occurs more commonly than previously reported and is more common in those who commence therapy at an older age. The combination of clinical symptoms, the temporal relationship to anti‐TNF therapy, and a panel of serological markers can assist clinicians in confirming a diagnosis.

## Introduction

The introduction of anti‐tumor necrosis factor alpha (anti‐TNF) medications, including infliximab and adalimumab, has revolutionized the management of patients with inflammatory bowel disease (IBD). These agents have demonstrated efficacy, are well tolerated, and have a relatively good safety profile.^1^ Pivotal clinical trials and postmarketing surveillance studies have identified some drug‐related adverse events, including infusion reactions, infections, and malignancy, as well as a number of immune‐mediated phenomena.^2^, ^3^, ^4^

Anti‐TNF medications have been demonstrated to induce autoimmunity in the form of antinuclear antibodies (ANAs) and anti‐double‐stranded deoxyribonucleic acid (anti‐dsDNA) antibodies. 5, 6 Pooled analysis of the initial open‐labeled clinical trials for infliximab in rheumatoid arthritis demonstrated an increase in ANA positivity from 29% pretreatment to 53% posttreatment.^6^ In this analysis, 14% of patients also developed anti‐dsDNA antibodies. Some patients may also develop clinical symptoms that mimic idiopathic lupus, and this has been recognized as a distinct clinical entity called anti‐TNF‐induced lupus (ATIL).^7^ The most widely accepted diagnostic criteria for ATIL are (i) a temporal relationship between symptoms and anti‐TNF therapy and resolution of symptoms following cessation of the offending medication; (ii) at least one serologic American College of Rheumatology (ACR) criterion of SLE, being either a positive ANA or anti‐dsDNA; and (iii) at least one nonserological criterion such as arthritis, serositis, or rash.

The estimated reported incidence of ATIL based on postmarketing studies is 0.19–0.22% for infliximab and 0.10% for adalimumab.^8^ The actual incidence, however, is difficult to determine due to lack of prospective studies, lack of recognition of the condition, and the overlap in clinical symptoms with the extraintestinal manifestations of IBD. Given the widespread use of anti‐TNF agents in the management of IBD, our aims were to evaluate the incidence and clinical and serological markers of ATIL in this population.

## Materials and methods

### *Study design and participants*

This study was a retrospective observational cohort study based on a prospectively collected registry of all IBD patients who were commenced on anti‐TNF therapy at Royal Perth Hospital, Western Australia between January 1st 2008 and December 31st 2017. The study population included all patients with a confirmed diagnosis of IBD, who were commenced on a course of anti‐TNF therapy, (infliximab or adalimumab), and had completed induction and at least one maintenance dose of therapy. Patient follow up occurred till a census date of 31st March 2018.

Patients were retrospectively defined as having ATIL based on the aforementioned diagnostic criteria: (i) a temporal relationship between symptoms and anti‐TNF therapy and resolution of symptoms following cessation of the offending medication (within 3 months of discontinuation); (ii) at least one serologic ACR criterion of SLE, being either a positive ANA or anti‐dsDNA; and (iii) at least one nonserological criterion such as arthritis, serositis, or rash.^7^ The incidence of ATIL and time to diagnosis were calculated for both infliximab and adalimumab. Demographic and clinical parameters, including age, gender, type of IBD, and use of concurrent immunomodulatory therapy, were obtained from electronic records.

Age, gender, and IBD type were evaluated as predictors in the development of ATIL. The results of several serological markers, if performed, including ANA, anti‐ds‐DNA, antihistone antibodies, anti‐Smith antibodies (anti‐Sm), anticyclic citrullinated peptide (anti‐CCP), and rheumatoid factor (RF), were also collected.

### *Statistical analysis*

Statistical analyses were performed using Stata (StataCorp 2015. Release 14) For the clinical and demographic predictors, continuous variables were analyzed using a two‐sided *t*‐test and categorical variables using a Fishers exact test or Pearson's *χ* ^2^ test. The threshold for statistical significance was defined as a *P* value < 0.05.

## Results

A total of 454 patient treatment courses with anti‐TNF therapy were included, comprising 300 with infliximab and 154 with adalimumab. Seventeen (5.7%) patients who received infliximab developed ATIL, and one patient (0.6%) on adalimumab developed ATIL. The clinical, demographic, and serological characteristics of each case of ATIL are summarized in Table 1. Twelve patients received glucocorticoids at the time of symptom manifestation. Patients who developed ATIL were more likely to be older at the time of commencement of anti‐TNF medication (46.47 years ± 13.79 years *vs* 38.85 years ± 14.75 years, *P* = 0.033) (Table 2). There was a trend that female gender was a risk factor for ATIL (72.2% *vs* 47.0%, *P* = 0.052). The median duration of therapy till development of ATIL was 15 months (range of 3–62 months).

**Table 1:** Sociodemographic, clinical, and serological characteristics of individual patients diagnosed with anti‐TNF‐induced lupus

Age	Gender	IBD	Anti‐TNF	Duration of therapy (months)	Immunomodulator	Clinical Symptoms	ANA (baseline) (<7 IU/mL)	ANA (at diagnosis) (<7 IU/mL)	dsDNA (<7 IU/mL)	Anti‐histone	Anti‐Smith	Anti‐CCP	RF (<14k U/L)
48	M	CD	Infliximab	21	Nil	Polyarthritis	0	30	38	Pos	Neg	NA	NA
69	F	CD	Infliximab	6	Nil	Polyarthritis	5	25	35	Neg	Neg	1	<10
34	F	CD	Infliximab	9	MMF	Rash	NA	30	NA	Neg	Neg	NA	NA
57	F	CD	Infliximab	10	Nil	Polyarthritis,	6	15	10	Neg	Neg	NA	<10
53	F	UC	Infliximab	3	Nil	Polyarthritis, Rash	2	10	0	Pos	Neg	1	11
51	F	CD	Infliximab	30	AZA	Polyarthritis	NA	30	NA	Neg	Neg	NA	NA
37	F	CD	Infliximab	9	AZA	Polyarthritis	2	30	NA	NA	NA	NA	NA
53	F	CD	Infliximab	10	MTX	Polyarthritis	0	30	22	Neg	Neg	NA	NA
39	M	CD	Infliximab	44	Nil	Rash	0	30	35	Neg	Neg	1	<10
62	F	CD	Infliximab	30	6MP	Polyarthritis,	7	30	6	Neg	Neg	NA	NA
36	M	UC	Infliximab	18	Nil	Rash	NA	25	6	Neg	Neg	NA	NA
49	M	CD	Infliximab	7	Nil	Polyarthritis	2	30	5	NA	NA	NA	NA
45	F	UC	Infliximab	33	Nil	Rash	7	30	7	Neg	Neg	NA	NA
21	M	CD	Infliximab	12	Nil	Polyarthritis	7	30	11	Neg	Neg	NA	<10
42	F	CD	Infliximab	21	Nil	Polyarthritis	5	30	39	Neg	Neg	5	<10
77	F	CD	Infliximab	3	Nil	Polyarthritis	NA	7	53	NA	NA	NA	<10
51	F	CD	Infliximab	28	Nil	Polyarthritis	2	30	6	Neg	Neg	2	<10
41	F	CD	Adalimumab	62	Nil	Polyarthritis Polyarthritis	0	30	52	Neg	Neg	NA	NA

Age–age at diagnosis of ATIL, Immunomodulator—concurrent at time of diagnosis.

AZA, Azathioprine; MMF, mycophenolate mofetil; MTX, methotrexate; 6MP, 6 mercaptopurine.

**Table 2:** Differences in characteristics of between ATIL and non‐ATIL

	Non‐ATIL (*n* = 436)	ATIL (*n* = 18)	*P* value
Male/female	231/205	5/13	0.052
Age			
Mean ± SD (years)	38.85 ± 14.75	46.47 ± 13.70	0.033
IBD type			
CD	312 (71.6%)	15 (83.3%)	0.209
UC	119 (27.3%)	3 (16.7%)	0.241
IBD‐U	5 (1.1%)	0 (0%)	0.816

All patients with a diagnosis of ATIL demonstrated an elevated ANA with results ranging from 7 to 30 IU/mL. In those patients who had a baseline ANA performed prior to commencing anti‐TNF therapy, the ANA level increased compared with their baseline level. Additional serological markers that were positive in patients diagnosed with ATIL included anti‐ds‐DNA in 10 of 15 (67%) and antihistone antibodies in 2 of 15 (13%.) Serological testing to exclude other inflammatory conditions, including idiopathic SLE and seropositive arthropathies, were performed in a subset of the patients (Table 3).

**Table 3:** Serological profile of patients diagnosed with ATIL

Autoantibody	Number (%)
ANA	18/18 (100%)
dsDNA	10/15 (66.7%)
Anti‐Histone	2/13 (15.4%)
Anti‐Smith	0/15 (0%)
Anti RF	0/8 (0%)
Anti‐CCP	0/5 (0%)

Five patients were on concurrent immunomodulator therapy at time of ATIL diagnosis. Ten patients on infliximab who developed ATIL were subsequently switched to adalimumab, and none of these developed ATIL on adalimumab, with a median follow‐up period of 29.3 months.

## Discussion

ATIL is a distinct clinical entity increasingly recognized and described in the rheumatologic literature. Data remain limited in the IBD population due to lack of recognition of the condition, as well as difficulty making a diagnosis due to significant overlap in symptoms with extraintestinal manifestations of IBD and other autoimmune diseases. Our study demonstrates an incidence rate of 5.7% for infliximab and 0.6% for adalimumab, which are much higher than postmarketing studies.^8^ This corresponds to a retrospective study in an IBD population, which identified 20 of 289 patients (6.9%) on anti‐TNF therapy who developed a lupus‐like reaction.^9^

This is a significant finding, given the widespread and increasing utilization of anti‐TNF therapy in IBD patients, as our results suggest that 1 in 20 patients who commence infliximab will develop ATIL. Infliximab is considered more immunogenic compared with adalimumab based on its chimeric structure and is also thought to reach higher tissue concentrations.^10^ Clinicians should counsel patients about the potential risk prior to commencing therapy and should monitor for clinical manifestations of lupus, which include rash, photosensitivity, and arthritis, as well as rare systemic manifestations, including pericarditis and neurological and hematological disorders, during routine follow up. 11 The extraintestinal manifestations of IBD include some of these symptoms, which make the diagnosis of ATIL more challenging. Peripheral arthritis can occur in both ulcerative colitis and Crohn's disease. It typically manifests as a nonerosive seronegative arthritis and occurs in between 5 and 10% of patients with ulcerative colitis and 10–20% of patients with Crohn's disease.^12^ Various cutaneous manifestations have also been associated with IBD and can occur in up to 15% of patients.^12^ There are various other autoimmune conditions, including the seropositive arthropathies and idiopathic SLE, which may occur in patients with IBD and also share clinical manifestations with ATIL. It is very difficult to differentiate ATIL and these various conditions based on clinical symptoms alone, and further information, including the temporal relationship with anti‐TNF therapy and serological markers is required.

Anti‐TNF therapies have been demonstrated to induce autoantibodies; however, no specific tests have been validated for ATIL. The most widely recognized diagnostic criteria require a positive ANA or ds‐DNA.^6^ All the patients with confirmed ATIL in our cohort had a positive ANA at the time of diagnosis, with an increase in the titer from baseline level. Anti‐ds‐DNA levels were elevated in 66.7% of patients tested. Anti‐Sm antibodies are part of the serological diagnostic criteria for SLE. The presence of these antibodies is almost exclusive to idiopathic SLE, and it is rarely found in drug‐induced SLE. 13 Antihistone antibodies have been found to occur in 95% of cases of drug‐induced SLE; however, this antibody is not pathognomonic as they are also found in 75% of patients with idiopathic SLE.^14^ The presence of antihistone antibodies in studies of patients with ATIL, however, report much lower rates, ranging from 17% to 57%.^13^ Only 15.4% of patients diagnosed with ATIL demonstrated positive antihistone antibodies in our cohort. A full panel of autoantibodies, including ANA, anti‐ds‐DNA, anti‐CCP, RF, antihistone antibodies, and anti‐Sm antibodies, should be performed in all patients with a clinical suspicion for ATIL.

We were unable to establish whether concurrent immunomodulatory therapy was a factor in the development or prevention of ATIL as patients in the cohort who did not develop ATIL were on both varying doses and durations of immunomodulator therapy. It has been postulated that concurrent immunomodulatory therapy may decrease the immunogenicity of a biologic therapy.^15^ Use of an immunomodulator, in the form of a thiopurine or methotrexate, when used together with a biologic agent has been associated with decreased formation of antidrug antibodies^16^ Further studies are required to assess whether concurrent immunomodulatory therapy reduces the development of ATIL. Based on small numbers, our study does suggest that it is safe to switch to adalimumab if a patient develops ATIL on infliximab.

Our study had several limitations. It's retrospective nature makes it prone to data entry error. As patients were retrospectively diagnosed with ATIL, the true incidence is likely underestimated as serology, which is a component of the diagnostic criteria, would only be performed if a diagnosis was suspected at the time, at the discretion of the treating physician.

ATIL is a complication of anti‐TNF therapy that is more common than previously reported. Diagnosis in an IBD population is often difficult due to lack of recognition of the condition and shared clinical symptoms with both the extraintestinal manifestations of IBDs and other autoimmune conditions. The combination of clinical symptoms, the temporal relationship to anti‐TNF therapy, and a panel of serological markers can assist clinicians in confirming a diagnosis.
